# Neurosurgery training in Nepal: then and now

**DOI:** 10.3389/fsurg.2023.1211722

**Published:** 2023-06-23

**Authors:** Bipin Chaurasia, Rupesh Raut, Raushan Chaurasia, Amit Thapa

**Affiliations:** ^1^ Department of Neurosurgery, Neurosurgery Clinic, Birgunj, Nepal; ^2^ Department of Neurosurgery, Patan Academy of Health Sciences, Patan, Nepal; ^3^ Department of Psychiatry, Psychotherapy and Psychosomatics, Hospital Herford, Herford, Germany; ^4^ Department of Neurosurgery, Kathmandu Medical College, Kathmandu, Nepal

**Keywords:** neurosurgery training, Nepal, residency, MCh, MS

## Abstract

**Introduction:**

Neurosurgery training lacks uniformity across the world. Differences in the methods of training adopted during the training period is one of the major problems encountered in the field of neurosurgery all over the world. Moreover, neurosurgery is not “one neurosurgery”; in other words, it is not a unified whole.

**Material and methods:**

In this study, we attempt to evaluate the present conditions of neurosurgery training in Nepal by exploring different institutions providing the training.

**Results:**

Due to various factors and challenges, the neurosurgery training provided in Nepal varies in different institutions. Many travel abroad for training due to a lack of sufficient seats in training institutions.

**Discussion and conclusions:**

Despite the challenges, the future is bright for neurosurgery training in Nepal. With continued investment in education and training, and the adoption of new technologies and techniques, it is likely that the field of neurosurgery will continue to thrive and make a positive impact on the health and well-being of the Nepali population.

## Introduction

Globally, training in neurosurgery lacks uniformity. Most parts of the world still lack neurosurgical services and training. The training period for neurosurgeons varies from 3 to 8 years in different countries; in Northern America, it is 7 years, and in the United Kingdom, it is 8 years.

Neurosurgery training in Nepal is currently available for those who have already completed 3 years of their residency in Master of Surgery (MS) in general surgery. Eligible candidates are required to take an entrance examination conducted by the Medical Education Commission (MEC), and if successful, they are allowed to pursue, for a further period of 3 years, a super specialization training program in neurosurgery in affiliated centers (Table 1). The university-affiliated program offers a Master of Chirurgiae (MCh) degree course in neurosurgery, and non-university training centers offer a National Board of Medical Specialties surgical specialty (NBMS-SS) course. These courses are designated as a Research Degree by the MEC of Nepal as both require candidates to submit a prospectively conducted research work. A fellowship postgraduate residency program called FCPS is also being conducted by the College of Physicians and Surgeons Pakistan (CPSP) in a few hospitals in Nepal. The total duration of this program is 5 years if one chooses subspecialties such as neurosurgery after the second year of training in general surgery. This program can be compared with other neurosurgery training programs in neighboring countries where students can enroll directly in 5- or 6-year training programs in neurosurgery after completing their undergraduate courses like MBBS or any other equivalent degree. These programs are called MS Neurosurgery (5 years of training) and Diplomate of National Board (DNB) Neurosurgery (6 years of training).

**Table 1:** Different institutions offering MCh/NBMS-SS training in neurosurgery in Nepal along with the number of new yearly intakes .

SN	Institutions	Course	Positions/year
1	College of Medical Sciences Teaching Hospital, Chitwan	MCh	1
	Chitwan Medical College, Chitwan	MCh	1
2	Kathmandu Medical College Teaching Hospital, Kathmandu	MCh	2
3	Maharajgunj Medical Campus (TUTH), Kathmandu	MCh	2
4	Manipal College of Medical Sciences, Pokhara	MCh	1
5	National Academy of Medical Sciences (NAMS), Kathmandu	MCh	2
6	Nobel Medical College Teaching Hospital, Biratnagar	MCh	1
7	Upendra Devkota Memorial National Institute of Neurological and Allied Sciences, Kathmandu (NINANS)	NBMS-SS	1

Both these training programs are currently unavailable in Nepal. Graduates with similar foreign degrees are currently working as neurosurgeons in several institutions/hospitals of Nepal after being certified by the Nepal Medical Council (NMC). Due to a limited number of seats in residency, many pursue their training in neighboring countries such as Bangladesh and Pakistan to obtain postgraduate degrees such as MCh, MS, and FCPS. Some even travel to Japan, China, and Russia for their PhD.

## Results

Since last year, the MEC has increased the number of seats in neurosurgery for trainees. Previously, this was done only in four institutions, but now, the total number of seats has been increased to 11 in seven different institutions (Table 1) (1). The other institutions that have been recognized by the MEC are national institute of neurological and allied sciences (NINANS), Bansbari, and Nepal Mediciti Hospital. But unfortunately, these two institutions did not get sufficient candidates because the number of candidates who passed the entrance examination was less than the available seats.

The MCh neurosurgery course was started in 2002 in the Department of Neurosurgery, Bir Hospital, under the affiliation of Kathmandu University (KU). In this course, there are two entry gates similar to those in all india institute of medical sciences (AIIMS): either direct entry after MBBS with 1-year exposure in neurosurgery or entry after an MS in general surgery, but with a different set of questions.. Then, the National Academy of Medical Sciences was established in Bir Hospital, and this institution started its own 3-year MCh program in 2010. Now, this has been taken over by the MEC.

## Discussion

In 2008, the Nepalese Society of Neurosurgeons (NESON) was formed with 17 members. Now, the number of this has increased to 120, with 44 being life members of this professional body. The NESON became a member of the World Federation of Neurosurgical Societies (WFNS) in 2010 (2).

Of the 3 years of training in MCh Neurosurgery, residents in the first year are mostly involved in ward chores, get trained in documentation, and attend theory classes as well as assist surgeons in the operating theater. By the time they reach the end of their training period, they are well experienced with different neurosurgical procedures and are allowed to operate on patients under supervision. The residents are encouraged to gain wide experience in the field of neurosurgery, ranging from neuro-trauma to neuro-oncology to different subspecialties of neurosurgery. During this period, the residents have opportunities to attend national and international conferences and present their papers of interest, which helps them to broaden their outlook. They participate in workshops and continuing medical education (CME) workshops and also visit other hospitals. The NESON has already included the category “best residents award” in its conferences to encourage residents.

However, even with almost 50,000 trained neurosurgeons worldwide, the ever-increasing cases of neurosurgical patients remain poorly attended (3–7). The major reason for this is the large volume of cases with fewer neurosurgeons in low- and middle-income countries (LMICs) (8–13). A comparison in terms of patient load and available neurosurgeons in Nepal at the time of writing reveals that Nepal lags far behind the Western world (14–17). However, the number of neurosurgeons per 100,000 population in Nepal is on a par with other South Asian countries such as India and Bangladesh. Currently, Nepal has one neurosurgeon for every 279,000 people. With the population growing at a rate of 1.85%, the country may need approximately 324 neurosurgeons in total. Presently, the annual increment in the neurosurgical workforce at the rate of 25 neurosurgeons per year still falls short of the target of providing for an adequate number of neurosurgeons over the next decade.

The experiences and expectations of the residents in residency programs in LMICs may be different from those of neurosurgery residents in residency programs in high-income countries (4, 18–22). In the West, residency training is being monitored with feedback commissioned independently from both instructors and trainees by central authorities such as the MEC and university officials. Although undoubtedly, Nepal is at the nascent stage of the training program, the country is rapidly progressing toward betterment. The NESON recognizes and addresses the deficiencies perceived by neurosurgery residents in their training program by conducting courses and workshops. NESON'S two subspeciality chapters like spine and vascular surgery chapters are playing active roles by conducting regular webinars,workshops, CME, meetings for trainees and enthusiastic neurosurgeons .In this way both trainee and trainers get opportunity to learn and interact with each other.

One other area where Nepal is lagging behind is fellowship training in subspecialties of neurosurgery. Interested neurosurgeons travel abroad for training. Recently, the NESON has started a fellowship program in spine surgery, called Fellow of NESON-Spine (FNS-Spine), for NMC-certified neurosurgeons. The fellowship is a structured program for over a year, which involves rotational training in various centers in Nepal and abroad. Given the fact that the number of fellowships has grown exponentially over the past decade, Nepal needs to train neurosurgeons in different subspecialties (5, 6, 23–25).

## Conclusions

Nepal still needs more trained residents to provide adequate neurosurgical care to its people. The government should take the initiative to increase the number of training seats in teaching and medical colleges so that neurosurgeons could be produced, rather than depending on foreign-trained neurosurgeons.

## References

[1] Seats of DM/MCh-Medical education commission. Available at: https://mec.gov.np/en/detail/seats-of-dmmch (Accessed December 19, 2022).

[2] History of NESON: NESON. Available at: https://neson.org/history-of-neson/ (Accessed December 19, 2022).

[3] Raut R. Neurosurgery across the globe. In: Agrawal N, editor. Surviving neurosurgery. USA: Springer (2021). p. 189–94.

[4] Deora H Garg K Tripathi M Mishra S Chaurasia B. Residency perception survey among neurosurgery residents in lower-middle-income countries: grassroots evaluation of neurosurgery education. Neurosurg Focus. (2020) 48(3):E11. 10.3171/2019.12.FOCUS19852 32114547

[5] Thapa A. Sub-specialty in neurosurgery: time has come for Nepal. NJNS. (2022) 18(3):1–2. 10.3126/njn.v18i3.39183

[6] Javed S Shabbir RK Khan T Yaqoob E Park KB Chaurasia B. Global neurosurgery: the Pakistani perspective. Neurosurgery. (2023) 92(2):e31–2. 10.1227/neu.0000000000002265 36637283

[7] Robertson FC Esene IN Kolias AG Kamalo P Fieggen G Gormley WB Task-shifting and task- sharing in neurosurgery: an international survey of current practices in low- and middle-income countries. World Neurosurg X. (2020) 6:100059. 10.1016/j.wnsx.2019.100059 32309800 PMC7154228

[8] Robertson FC Esene IN Kolias AG Khan T Rosseau G Gormley WB Global perspectives on task shifting and task sharing in neurosurgery. World Neurosurg X. (2020) 6:100060. 10.1016/j.wnsx.2019.100060 32309801 PMC7154229

[9] Mukhida K Shilpakar SK Sharma MR Bagan M. Neurosurgery at Tribhuvan University Teaching Hospital, Nepal. Neurosurgery. (2005) 57(1):172–80. 10.1227/01.NEU.0000163429.92507.D4 15987553

[10] Agrawal A Kumar A Agrawal CS Pratap A. One year of neurosurgery in the eastern region of Nepal. Surg Neurol. (2008) 69(6):652–6. 10.1016/j.surneu.2007.03.047 18486707

[11] Sapkota S Rijal S Karn M. Neurosurgery in Nepal: past, present, and future. World Neurosurg. (2022) 158:100–5. 10.1016/j.wneu.2021.10.162 34728395

[12] Kato Y Liew BS Sufianov AA Rasulic L Arnautovic KI Dong VH Review of global neurosurgery education: horizon of neurosurgery in the developing countries. Chin Neurosurg J. (2020) 6(03):178–90. 10.1186/s41016-020-00194-1 PMC7398343 32922948

[13] Shilpakar SK. Subspecialties in neurosurgery and its challenges in a developing country. World Neurosurg. (2011) 75(3–4):335–7. 10.1016/j.wneu.2010.12.047 21600457

[14] Munakomi S Bajracharya A. Reappraising neurosurgical residency in Nepal—stepping stone for paradigm shift from its current perspectives. World Neurosurg. (2020) 133:8–9. 10.1016/j.wneu.2019.09.030 31610426

[15] Roka YB. Subspecialties in neurosurgery: are we ready? Nepal J Neurosci. (2018) 15(1):1–2. 10.3126/njn.v15i1.20015

[16] Hoffman C Härtl R Shlobin NA Tshimbombu TN Elbabaa SK Haglund MM Future directions for global clinical neurosurgical training: challenges and opportunities. World Neurosurg. (2022):e404–e18. 10.1016/j.wneu.2022.07.030 35868506

[17] Thapa A. Adversity quotient in neurosurgical training. Nepal J Neurosci. (2020) 17(2):1–3. 10.3126/njn.v17i2.30109

[18] Kanmounye US Mbaye M Phusoongnern W Moreanu MS Niquen-Jimenez M Rosseau G Neurosurgical training in LMIC: opportunities and challenges. In: Ammar A, editor. Learning and career development in neurosurgery: values-based medical education. New York: Springer, Cham (2022). p. 219–27.

[19] Sader E Yee P Hodaie M. Assessing barriers to neurosurgical care in sub-Saharan Africa: the role of resources and infrastructure. World Neurosurg. (2017) 98:682–8. 27506409 10.1016/j.wneu.2016.07.102

[20] Roka YB. Neurosurgery in eastern Nepal: the past, present and future and a near decade of personal experience. Nepal J Neurosci. (2017) 14(2):1–2. 10.3126/njn.v14i2.19696

[21] Parajuli S Gyawali P Subedi P Bhatta OP Mukhia R Sedain G Neurosurgery as a career choice among recent medical graduates from the Institute of Medicine, Nepal: a cross-sectional study. Nepal J Neurosci. (2022) 19(4):39–42. 10.3126/njn.v19i4.44702

[22] Neupane D Dahal A Lageju N Jaiswal LS Pandit N Mahat A Letter to the editor regarding “neurosurgery residency education in the post- coronavirus disease 2019 (COVID-19) era: planning for the future”. World Neurosurg. (2023) 172:103. 10.1016/j.wneu.2022.12.045 37012709 PMC10060018

[23] Thapa A. Neurosurgical training: adapting to the new normal. Nepal J Neurosci. (2020) 17(3):1–3. 10.3126/njn.v17i3.33094

[24] Drummond KJ Kim EE Apuahe E Darbar A Kedia S Kuo MF Progress in neurosurgery: contributions of women neurosurgeons in Asia and Australasia. J Clin Neurosci. (2021) 86:357–65. 10.1016/j.jocn.2021.02.002 33618964

[25] Chaurasia B. The expanding universe (of neurosurgery). Oper Neurosurg. (2022) 11:10–227. 10.1227/ons.0000000000000692 36929764

